# Review of *radiomics and radiogenomics* and *big data in radiation oncology*

**DOI:** 10.1002/acm2.12955

**Published:** 2020-07-09

**Authors:** Dandan Zheng

**Affiliations:** ^1^ University of Nebraska Medical Center Omaha NE USA

## 1 Overview

Medical big data science research such as radiomics has soared in recent years and found many potential applications in medical physics. In contrast to the tremendous interests in these exciting new directions, comprehensive learning materials and books have been scarce on these topics, especially those that tailor toward the radiation oncology and radiology communities. Despite an abundance of research papers and some review articles, there have not been many comprehensive books devoted to these special audiences. Two first‐edition books published in 2019 by the Taylor and Francis Group, *Radiomics and Radiogenomics* (edited by Ruijiang Li, Lei Xing, Sandy Napel, and Daniel L. Rubin) and *Big Data in Radiation Oncology* (edited by Jun Deng and Lei Xing), have opportunely filled this void, and provided a comprehensive review as well as valuable insights on these key new advances. The chapter authors for the two books are reputable experts on related research areas, including many leading figures such as Robert Gillies, Maryellen Giger, Joseph Deasy, Issam Naqa, Laurence Court, and Charles Mayo. The books belong to an ongoing book series titled “Imaging in Medical Diagnosis and Therapy” that was first initiated over a decade ago by a group of senior leaders in the medical physics community, with each volume to address “a rapidly advancing area of medical imaging or radiation therapy of importance to medical physicists”.

## 2 Purpose and Audience

Since both books relate to data science, and radiomics is an application of big data in radiation oncology, the two books have slight topic overlaps, while still having distinct focuses and addressing somewhat different audiences.

*Radiomics and Radiogenomics* seeks to cover the fundamental principles, technical basis, and clinical applications of radiomics and radiogenomics, with a focus on oncology. In this context, radiomics is defined as the discovery of imaging biomarkers with potential diagnostic, prognostic, or predictive value; and radiogenomics is the identification of molecular biology behind these imaging phenotypes. The intended audience of the book includes imaging scientists, medical physicists, radiologists, radiation oncologists, and other related researchers and clinicians. The authors have successfully achieved their goal of providing a comprehensive review of the field, including a detailed understanding of the technical development and clinical relevance and a grounded appreciation of state‐of‐the‐art technology and future directions.

*Big Data in Radiation Oncology* focuses on an in‐depth yet broad discussion of how the big data accumulated in radiation oncology clinics is impacting and will continue to impact radiotherapy practices. In addition to radiomics and radiogenomics, big data applications also extend to treatment planning, organ dose tracking, comparative effectiveness, cancer registry, outcome predicting models, and multiparameterized models etc. The book is therefore targeted toward a wide audience related to radiation oncology such as physicians, physicists, dosimetrists, healthcare practitioners, regulatory bodies, insurance companies, and industrial stakeholders, in addition to data scientists and biostatisticians.

Data science is a rapidly advancing field and plays an important and active role in reshaping the clinical practice of radiology, radiation oncology, and medical physics. Although these two books were not specifically written as textbooks, they cover sufficient breadth and depth to serve as such. Because of the current lack of such textbooks, they could be helpful resources for training residents and graduate students on these topics, in addition to being a handbook for new researchers in the field.

## 3 Contents and Highlights

*Radiomics and Radiogenomics* is approximately 400 pages in length and contains 22 chapters. After an introductory chapter, the main contents of the book are organized into two parts, a Technical Basis part focusing on the technical basis and resources to support radiomics and radiogenomics research (Chapters 2–11), and a Clinical Applications part devoted to summarizing current clinical applications in oncology (Chapters 12–20). At the end, two chapters briefly discuss emerging research directions and future outlooks including a roadmap to clinical translation. For the Technical Basis section, individual chapters include those related to different imaging modalities (CT, PET/CT, and MRI), and those related to the major steps of radiomics workflow (segmentation, feature extraction, and predictive modeling). There are also single overview chapters for the topics of imaging informatics, MRI habitat imaging, rationale and methods for radiogenomics, and very usefully, radiomics resources and datasets. The Clinical Applications section is organized by anatomical disease sites, including brain, breast, lung, head and neck, GI, GU, and GYN. It also includes an overview chapter of “pathways to radiomics‐aided clinical decision‐making” and, interestingly, a chapter of “applications of imaging genomics beyond cancer”—which includes clinical applications for neurological and psychiatric disorders where brain imaging has traditionally played an important role.

*Big Data in Radiation Oncology* is 289 pages in length and contains 16 chapters. The book is well organized into four main groups: Basics (overview), Techniques (data standardization, storage and databases, machine learning, cloud computing, and statistical methods), Applications (treatment planning, quality assurance, organ dose tracking, comparative effectiveness research, cancer registry, radiogenomics, and radiomics), and Outlooks (clinical and cultural challenges, future perspectives on outcome modeling, early cancer detection, and prevention). Radiation oncology is a medical field uniquely suited for big data analytics because treatment planning and delivery data are very structured. However, the analytics use of this type of data has thus far been rare and often limited to a single institution. Therefore, the chapters of this book offer much needed discussions on this important topic, giving excellent fundamental information, and delineating challenges and solutions.

## 4 Critical Assessment

Big data research in radiation oncology and radiology, including radiomics, is an area that has attracted increasing research and development in the past decade, especially within the past 5 yr. There has been an explosive production of literature on these topics and still even more studies continue to be conducted. Yet there has been a lack of published texts that comprehensively discuss these areas, partially due to recentness and the ongoing rapid evolution of the fields. These two books, with the breadth and depth of the information they provide, are very timely. Published in 2019, the books also list important references in each chapter so the readers can easily pursue the topics more deeply.

I highly recommend these two books for current or new researchers in relevant fields, both as a handbook for technical details, and as an inspiring read to gain a global view of the fields and spark new ideas. From the technical viewpoint, the two books provide researchers with very good information on data and process standardization that facilitates proper study design. For example, *Radiomics and Radiogenomics* has a thorough discussion about the uncertainties involved in the steps of radiomic feature computation and predictive modeling, as well as mitigation strategies. Currently, many research papers have passed peer review and appeared in journals, but still contain design flaws which ultimately limit the robustness and hence the applicability of the developed models. From these two books, readers can gain a fundamental understanding of radiomic feature definition and computation, processing steps (such as voxel resampling, MRI field bias correction and normalization, and other data harmonization), and processing parameters (such as fixed bin size vs fixed bin number and voxel neighborhood size). Readers can also develop a deeper appreciation of proper data management in modeling from both texts. As such, the technical knowledge from the books can assist researchers in optimizing their own study design. The “Resources and Datasets for Radiomics” chapter of *Radiomics and Radiogenomics* is also especially helpful as it contains a comprehensive list of currently available software and datasets, as well as an excellent discussion on the repeatability and reproducibility of radiomics. From the application viewpoint, the books also offer a comprehensive picture of the current state‐of‐the‐art clinical applications in these fields for the researchers to build their future investigations upon.

These books also work well for clinicians and other stakeholders to gain a broad, yet in‐depth insight into these fields and the applications that our clinical practice is quickly moving toward. Reading the books can help clinicians better understand the strengths and limitations of the current clinical implementations of these methods, as well as enabling them to knowledgeably appraise any future applications. In *Radiomics and Radiogenomics*, both the imaging modality chapters and anatomical site chapters provide an excellent status report on the current successes as well as challenges for the readers. *Big Data in Radiation Oncology* is especially suited for this usage, as it covers many different current applications in clinical radiotherapy and hence offers much needed comprehensive knowledge for clinicians in the age of big data and artificial intelligence. For example, I especially enjoyed reading the “Cancer Registry and Big Data Exchange” chapter and the “Cloud Computing for Big Data” chapter. Currently, dataset size is often still a limiting factor for single‐institution big data research in radiation oncology. Providing thorough discussions on big data exchange architectures and storage/processing solutions, these chapters give readers excellent information regarding the paradigm shift from centralized to distributed learning that could allow radiation oncology big data to be more scalable. Another “Clinical and Cultural Challenges” chapter further explores the current challenges such as patient privacy, ethical and social concerns, and limitations in current solutions, providing an interesting and informative read. The “Machine Learning for Radiation Oncology” gives a concise overview of machine learning methods, discusses some key concepts, and uses one real‐world example to illustrate differences.

Data science training has undoubtedly become increasingly important in the fields of medical physics, radiation oncology, and radiology. Therefore, these two timely books are also well suited as textbooks to train future researchers and clinicians in schools and residencies. However, as these two books are written more as topical reviews instead of pedagogic texts, there are no accompanying exercises or problem sets with the chapters. Educators will need to develop those independently for their teaching. In terms of contents, *Radiomics and Radiogenomics* would work well for courses such as radiomics and quantitative medical imaging; *Big Data in Radiation Oncology* would work well for a general, introductory or overview data science course in radiation oncology. For the latter, there is another available book entitled *Machine Learning in Radiation Oncology* by Naqa et al (Springer, 2015). While both lacking an exercise portion as a text book, comparing the two, the “Machine Learning” book focuses more on technical details and applications using machine learning, and the “Big Data” book is more generally related to big data, also covering other topics such as statistical methods, data storage, cloud computing, and data sharing. Outside of the medical physics focus, there are also other available generic texts on big data science.

Since the fields are rapidly evolving, it will be impossible for any book to be all‐inclusive. The two books have done an excellent job providing comprehensive and in‐depth discussions on the topics. In addition to the solid knowledge foundations the books lay on these topics, I also enjoy the futuristic perspectives provided by the experts in the outlook chapters and sections. There are a couple of topics that I wish were given more emphasis in the books. The first relates to the synergy of radiomics (or more generally, artificial intelligence in medical imaging) and other “‐omics” technologies, in terms of data integration and clinical applications. And the other is how the fields are adapting to and evolving with technological advances such as newer imaging scanners and reconstruction techniques, fundamentally new machine learning techniques such as the capsule network, and new biomarkers from other fields. On the other hand, these and/or many other new topics will most certainly be addressed in future editions of these books or similar future books, as the fields evolve.

Despite having been written independently by a large group of authors, the chapters of the two books are well organized and consistent to a large extent. Nevertheless, there are some content overlaps among different chapters. For example, in *Radiomics and Radiogenomics,* many chapters discuss the radiomics workflow or machine learning from scratch, making each chapter complete when standing alone but slightly redundant when read together. Regarding consistency, the “Quantitative Imaging using MRI” chapter of *Radiomics and Radiogenomics* bears relatively less relevance to radiomics compared to its counterpart chapters on CT and PET/CT, as it largely discusses preradiomics quantitative applications. It therefore serves as an excellent read on advanced MRI but is somewhat lacking for MRI radiomics.

In summary, these two books are timely comprehensive data science texts for the medical physics and related clinical/research communities. While best suited for current or new researchers in the fields and readers wanting an in‐depth overview of the topics, the books are also suited for a broad audience with clinical or regulatory interests, or as textbooks for student and resident training.

